# Case study: biosimilar anti TNFalpha (Adalimumab) analysis of Fc effector functions

**DOI:** 10.1186/1753-6561-7-S6-P30

**Published:** 2013-12-04

**Authors:** Carsten Lindemann, Silke Mayer, Miriam Engel, Petra Schroeder

**Affiliations:** 1 EUFETS GmbH, 55743 Idar-Oberstein, Germany

## Background

For the development of biosimilar monoclonal antibodies or related substances containing the IgG Fc part it is mandatory to fully compare immunological properties between originator and biosimilar in a "comparability exercise" [1]. The important Fc associated functions to mediate antibody dependent cellular cytotoxicity (ADCC) and complement dependent cytotoxicity (CDC) need to be characterized using both the active substance of the biosimilar and the comparator [2,3]. For testing anti TNFalpha antibodies target cells with stable expression of membrane TNFalpha (mTNFalpha) is required. Further prerequisites are test systems facilitating analysis with high precision and accuracy.

## Materials and methods

We generated a human transgenic NK-cell line (YTE756.V#26, effector cell line) with stable expression of Fc gamma-receptor IIIA (CD16, high affinity variant, valine at position 159) and stable functional characteristics to replace primary effector cells in ADCC assays. Target cells for ADCC and CDC assays were genetically modified for stable expression of mTNFalpha without the capability to release soluble TNFalpha. Both target and effector cells were generated using retroviral vectors to facilitate high and stable transgene expression. Vector particles were generated by transient transfection of 293T cells with plasmids encoding gag, pol/env and an expression plasmid containing the packaging region and the sequences of promotor and the transgenes, i.e. selection marker and gene of interest. Multiple gene expression was achieved either by using a bicistronic design enabling transcription from two promotor sequences, or by using an internal ribosomal entry site. Transduction of cells in log phase was followed by a selection of transduced cells and clonal selection by limiting dilution. Cell clones were expanded for primary and secondary cell banks and further characterised with regard to transgene expression and functional characteristics.

The more complex ADCC assays were developed employing design of experiments (DoE). To show assay suitability goodness of fit, ratio of upper to lower asymptote, slope and parallelism was determined for each dose-response curve compared to a standard. Hypo- and hyperpotent samples (50%, 100%, 150% and 200% potency) of Adalimumab and Infliximab were analysed in both ADCC and CDC assays to determine accuracy and linearity of each method.

For ADCC assays HT1080 mTNFalpha+ cells were seeded into 96-well plates 18 -20 h before start of the assay. Anti TNFalpha dilution series were performed in separate plates and transferred into the assay plate together with YTE756.V#26 effector cells at an E:T ratio of 10:1 using the effectors cell medium as assay medium. After an incubation time of 17 ± 1 h effector cells were washed from the adherent target cells. Quantification of residual target cells was performed by staining with XTT and photometric measurement. Each assay consists of standard (the biosimilar) and sample (originator) concentrations ranging from 1000 to 4.69 ng/ml in duplicates. Comparison of dose-response curves in a 4 PL model and determination of potency was performed using PLA software (Stegmann Systems).

For CDC assays CHO mTNFalpha+ cells were seeded into 96 well plates 20 - 25 h before start of the assay. Antibody dilution series were transferred into the assay plate using cell culture medium containing 20% native human serum pool. After an incubation time of 2 ± 0.5 h medium non-adherent cells were removed by washing the MTP. Quantification of residual cells was performed as described for ADCC assays. Each assay consists of standard and sample (originator or accuracy item) concentrations ranging from 5000 to 130 ng/ml in duplicates. Comparison of dose-response curves in a 4 PL model and determination of the relative potency was performed using PLA software.

Originator batches and the biosimilar were analysed by monosaccharide and sialic acid analysis, N-glycan profiling by MALDI-MS (permethylated glycans) and by HILIC-HPLC. N-Glycosylation site determination was done by MALDI and/or LC-ESI-MS and MS/MS (1 digestion).

## Results

Both ADCC and CDC assays show good accuracy (relative accuracy < 15%) and linearity (r squared < 0.97). Precision of CDC assays (CV < 8%) was better than that of the more complex ADCC assays (< 15%). Due to the distinctly lower actitivity of Adalimumab compared to that of Infliximab we evaluated the most influential factor for gaining a high asymptote ratio by DoE. The incubation time was shown to be most important compared to other factors as effector to target cell ratio and fetal bovine serum content.

We analysed different batches of originators and a biosimilar candidate molecule for functional variability in ADCC and CDC assays (Table 1). In CDC assays (n = 3) the three originator batches of Adalimumab showed comparable potency in between batches and compared to the biosimilar. A higher variability of the originators was found in ADCC assays (n = 6) besides the potency was higher than that of the Adalimumab biosimilar.

**Table 1:** Relative potency (compared to biosimilar) of originators in ADCC and CDC assays

Assay	Originator	Relative potency	CV
ADCC	2	140%	9.9%
	3	141%	11.1%
	4	135%	16.8%

CDC	2	92%	15.1%
	3	89%	10.2%
	4	89%	16.7%

Major differences between originators with regard to glycosylation were not found. The biosimilar showed a high galactose content and consequently a higher percentage of galactosylated glycan structures than the originators.

## Conclusions

In summary we show the suitability of an ADCC potency assay for investigation of functional comparability of Adalimumab and biosimilar candidate substances. Differences between biosimilar and originators in glycosylation might contribute to differences found in the ADCC potency assay but not with the CDC potency assay.
